# Parents' Perceptions of Delays and Disabilities in Their Children Requiring Invasive Mechanical Ventilation

**DOI:** 10.1002/ppul.71481

**Published:** 2026-02-09

**Authors:** Amanda Calipo, Emma L. Green, Iris Huang, Sarah A. Sobotka

**Affiliations:** ^1^ The University of Chicago Pritzker School of Medicine Chicago Illinois USA; ^2^ Section of Developmental & Behavioral Pediatrics, Department of Pediatrics The University of Chicago Chicago Illinois USA; ^3^ Department of Pediatrics University of California San Francisco San Francisco California USA

**Keywords:** autism, developmental delay, prematurity, tracheostomy

## Abstract

**Objective:**

To assess parents of children requiring invasive mechanical ventilation's (IMV) perceptions of their children's developmental delays and disabilities.

**Methods:**

Parents of children <3 years of age who required IMV after neonatal disease were interviewed as a consecutive series from a state‐wide agency (*n* = 20) and a ventilator clinic (*n* = 15). Interview topics included parents’: (1) perception of their child's current developmental functioning, (2) understanding of their child's disability risks, and (3) prior conversations with providers about developmental milestones and disability risks. Interviews were coded using a modified template approach and discussed to consensus. Main and sub‐themes were determined iteratively with all investigators.

**Results:**

Thirty‐five parents were interviewed. Themes were categorized under two topics: (1) Parents’ recall of conversations with providers; (2) Parents’ perspectives on developmental delays. Topic 1 themes: (1) Despite wanting information on developmental outcomes, parents report unclear expectations for delay and disability based on limited conversations; (2) Parents reported a lack of access to neurodevelopmental expertise, most often directing questions to therapists or other parents. Topic 2 themes: (1) Parents can identify specific developmental milestones and delays; (2) Parents have an idea of overall disability, sometimes attributed to specific diagnoses or medical complexity; (3) Parents are hopeful for their child's future development.

**Conclusion:**

Parents of children requiring IMV value honesty about disability and recall few prior conversations. Most parents can identify developmental delays yet expect catch‐up. Ultimately, improving communication between providers and parents about disability risk is critical to support children requiring IMV.

**Abbreviation**
IMV: invasive mechanical ventilation

## 1 Introduction

Children born prematurely who have severe neonatal lung disease requiring invasive mechanical ventilation (IMV) are often hospitalized for many months before going home. As per guidelines put forth by the American Thoracic Society [1], training for parents of children requiring IMV is considered an essential step towards discharge, and is often extensive, lasting weeks or months, and involving many interdisciplinary hospital providers. The majority of this training and care focuses on medical issues such as management of medical technologies (e.g. ventilators, feeding tubes), medication administration, and emergency preparedness [2, 3, 4]. Despite the extensive training, parents still describe steep learning curves between in‐hospital and home environments [5]. These gaps in preparation may be in part due to inpatient clinicians’ lack of experience with the post‐discharge realities of caring for children requiring IMV [6], and often little overlap between inpatient and outpatient care teams.

Despite the length and intensity of training with families, it is not known if families of children requiring IMV also receive counseling on risks of long‐term disability and developmental outcomes. It is well established that children born prematurely have high rates of disabilities across developmental domains: intellectual disability, autism spectrum disorder, cerebral palsy, learning disabilities, behavioral disorders, and emotional disorders [7, 8, 9, 10]. Even higher rates of developmental disability and delay have been identified in children born prematurely with worse chronic respiratory disease indicated by bronchopulmonary dysplasia [11, 12, 13], pulmonary hypertension [14, 15] and/or tracheostomies [16, 17, 18, 19]. Prior work by our study team has demonstrated that children with the severest form of neonatal lung disease requiring IMV have developmental delay across developmental domains, and when followed into toddlerhood, manifest higher rates of severe neurodevelopmental disability than previously published [20, 21].

Little is known about the disability‐risk counseling parents of children requiring IMV receive before hospital discharge and parents’ perceptions of the disability and developmental profiles of their children. Therefore, through qualitative interviewing, we sought to understand parents of children requiring IMV's (1) experiences with anticipatory guidance about developmental trajectory and disability risk, and (2) perceptions of the current developmental abilities of their children and expectations for future development and disability. The ultimate goal of this study is to understand current practices and parent perspectives in order to inform counseling.

## 2 Methods

### 2.1 Study Design and Recruitment

We recruited parents of children requiring IMV under age 3 at time of consent whose child resided at home and if available, both parents were invited to participate as a consecutive series from (1) a state‐wide agency between February 2019 and January 2022 (*n* = 20) and (2) an interdisciplinary ventilator clinic between September 2022 and April 2024 (*n* = 15). We recruited the first cohort for a primary study of hospital‐to‐home transition and developmental trajectories of young children requiring IMV. We recruited the second cohort for this study specifically focused on understanding developmental trajectories. The sample size of this second cohort was determined when new interviews no longer produced novel themes for the analysis of this study objective. Ultimately the combined dataset included interviews from 35 families with 50 participating parents. All parents were interviewed by the lead study investigator (SS), a clinical specialist in childhood disability, and expert in disability profiles of children with medical complexity. Interviews were conducted exclusively for research purposes and our study team did not provide clinical care to these children.

Families recruited through the state‐wide agency were screened for eligibility by the agency and referred to our study team. Parents with English or Spanish fluency were eligible; Spanish interpreter services were used with two participants. Parents were interviewed twice in the family home (or on Zoom during the COVID‐19 pandemic) and both interviews were included in the dataset for this analysis.

Families recruited through the interdisciplinary ventilator clinic were screened for eligibility by the clinic's nurse practitioner and then referred to study staff to be approached for recruitment, consent and enrollment activities in the clinic space. Video Spanish‐language translation was available as needed. All bilingual interviews were transcribed by a Spanish‐speaking professional transcriber.

Detailed methods for the initial interviews with families recruited through the state‐wide agency have been described previously [4, 20]. Data from the follow‐up interviews 6 months after the initial interview, and interviews from parents recruited from the interdisciplinary ventilator clinic have not been previously published.

All participating parents completed informed consent, and the projects were approved by the Institutional Review Board at the University of Chicago (IRB 17‐0908 and IRB 21‐1075).

#### 2.1.1 Sociodemographic & Medical Characteristics

All parents completed demographic and medical complexity surveys using Research Electronic Data Capture system [22]. Detailed information about medical diagnoses and number of subspecialist physicians seen were included in the survey in order to capture clinical complexity. Diagnoses were collapsed into common categories (e.g. congenital heart condition, genetic duplication or deletion) to maintain participant anonymity. Neighborhood socioeconomic disadvantage was measured by the National Area Deprivation Index (NADI) [23], calculated using a 9‐digit zip code. The NADI takes into consideration neighborhood context which has been associated with certain negative health outcomes. In the analysis of income, responses were dichotomized into 50,000 USD or less to protect participant anonymity in a small sample.

#### 2.1.2 Parent Semi‐Structured Interview

Semi‐structured interviews were conducted with the parents of children requiring IMV to understand their views of their child's development and prior counseling they received from providers about disability trajectories. Examples of topics covered include (1) Overall perception of child health and development; (2) Developmental areas of greatest concern; (3)Future outlook for child's development; (4) Recall of prior conversations with providers about delay and disability. The interview guide was tested prior to data collection with parents of children requiring IMV and modified in response to feedback solicited from experts in the field. The subsequent interview guide for parents from the ventilator clinic cohort was influenced by analysis of the state‐wide cohort to deepen understanding of main themes. Data from conversations during interview guide testing were not collected or included in the dataset. All parent interviews were audio‐recorded and professionally transcribed with identifiers redacted.

### 2.2 Data Analysis

All study authors were involved in coding: two medical students, one clinical research coordinator with 7 years of research experience with this population and additional expertise as a sibling of an adult requiring IMV, and the principal investigator who is a developmental‐behavioral pediatrician with expertise in children requiring IMV. During initial coding, the interview guide influenced our creation of a priori themes in the first draft of the codebook using a modified template analysis approach [24]. Each interview was coded independently by two reviewers using an open coding technique and after each interview was discussed to consensus, the codebook was iteratively updated. Data management was completed using MAXQDA 2022 software (VERBI Software, 2022) [25]. Codes related to the study objective were reviewed and initial themes identified. Data analysis continued until all study authors reached consensus about final themes and sub‐themes with supporting data. Common themes from the interviews were collated and classified with specific quotes to demonstrate themes and subthemes. The reflexive thematic analysis reporting guidelines (RTARG) and the Standards for Reporting Qualitative Research (SRQR) were used for reporting this study [26, 27].

## 3 Results

### 3.1 Participant Characteristics (Table 1)

Thirty‐five interviews with parents of children with IMV were conducted. Interviews lasted median (range) duration of 51.0 (16–149) min. The children had a median (range) age of 13 (6–40) months at enrollment and a median gestational age of 27 weeks (54.3% were extreme preterm, 14.3% very preterm, 11.4% moderate preterm, and 20% term). All children required gastrostomy tubes in addition to a tracheostomy and a ventilator. Most of the primary parents interviewed were mothers (97%) and most families reported an annual household income of <$50,000 (54%). The NADI median (range) was 52.6 (13–96).

**Table 1:** Characteristics of parents and children at enrollment (*n* = 35).

Demographic characteristics	*n*(%)
*Child Characteristics*	
Male child	17 (48.6)
Race/Ethnicity of Child	
Non‐Hispanic White	9 (25.7)
Non‐Hispanic Black	19 (54.3)
Hispanic	7 (20)
Age of child at enrollment in months median (range)	14.6 (5.6–40.1)
Diagnoses	
Prematurity	
Extreme (< 28 weeks)	19 (54.3)
Very (28–32 weeks)	5 (14.3)
Moderate to Late (32–37 weeks)	4 (11.4)
Term	7 (20)
Congenital heart condition	11 (31)
Genetic duplication or deletion	4 (11.4)
Other genetic anomaly	3 (8.6)
Number of subspecialists (median (range))	6 (1–12)
Feeding tube	35 (100)
Tracheostomy	35 (100)
Ventilator	35 (100)
*Parent/Family characteristics*	
Mother	34 (97.1)
Father	17 (48.6)
Marital Status	
Married	19 (54.3)
Single	11 (31.4)
Other	5 (14.3)
Household Income <$50,000*	19 (57.6)
National Area Deprivation Index median (range)	52.6 (13–96)
Insurance status	
Private only	1 (2.9)
Medicaid only	32 (62.9)
Both Medicaid and Private	12 (34.3)
Work Status**	
Full‐time or part‐time	16 (47.1)
Not currently working	18 (52.9)
Changes in work status due to care of child*	
No change in work hours	11 (33.3)
Decrease in work hours	22 (66.7)

* Two parents declined to respond;

** One parent declined to respond.

#### 3.1.1 Themes

Analysis of the interviews was separated into two main topics: (1) Parents’ Recall of Conversations about Delay/Disability and (2) Parents' Perspectives on Development and Delay.

*Main Topic I: Parents’ Recall of Conversations about Delay/Disability* (Table 2)

**Table 2:** Topic 1—Detailed quotes from parents of children requiring invasive mechanical ventilation (IMV) recalling conversations about delay and disability (*n* = 35).

Theme 1. Despite wanting the information and valuing developmental outcomes, parents report unclear expectations for delay and disability based on limited conversations with providers.	
1a. Parents report limited prior discussions about their child's long‐term development and disability risks, often because inpatient conversations were focused on acute medical conditions.	“We haven't had…anyone to even talk about the development… while he was in the hospital…everybody was telling me they're concerned about his lungs… and we'll get to the point where we have to worry about his development.”—C2F5
	“The only thing that they were really just worried about was her breathing, eating, tolerating feeds, stuff like that.”—C2F13
1b. Most parents describe wanting to know about their child's risks of long‐term disabilities.	“I just would like for people to kind of shoot it straight…be honest. Like, “Okay, well, normally we're seeing more.”… If we are heading down a road of some other sorts of things, so that we know that. So that then we're not surprised later.”—C1F1
	“Even if it's not going to happen, at least I know that there was a chance, and now I at least can prepare to deal with some of the things that might happen. Even if it doesn't happen—that would be a stress reliever. But I feel like I'd rather just know that there's potential.”—C2F2
1c. Parents do not have clear expectations for their child's developmental trajectory	“I feel like the one thing that's just so hard is like all the milestones, and the evaluation of like how is she doing…I'm like, “How do you measure that?” And then it's like they give you kind of a range, and you're like, “Well, is that—are a lot of preemies at that point at this point? Or a lot of babies with this sort of situation?” I just wish there was more to—that then you could be like, “Okay, yeah, this is pretty normal.” Or, “This is not.”—C1F15
	“They tell me that she's behind on some things, but they say that she's doing really great. I just don't know what that means. they keep saying she'll follow her own curve…I just don't know… will she live a normal life? Will she go to school? Will she be independent? I wish I knew more…Will she catch up? Like based on other kids that you have seen like her, when do they catch up? And I know it's hard to answer that, but how far behind is she?”—C2F8
	“I would like to know more about, what does this look like long term? And where is [child's name] at in that process?”—C1F11
1d. Parents who recalled developmental conversations with providers described them as vague and/or suggesting catch‐up potential.	D: Even the medical director at [Children's Hospital] he kind of assured us. He's like, “Listen.” He said to me, “Her lungs will develop. She will—she's developmentally…” She's actually…
	M: She's OK developmentally.
	D: She's actually OK.—C1F1
	“Basically, they're really just saying the same thing. He is delayed. But when he get older, he still might be a little behind, until he catches all the way up. I don't know how long that will be. It really depends on [child's name].”—C2F5
	“They said that since she had brain bleeds …that there would be potential signs of disability, but they weren't sure which kind, that they would have to keep waiting until she gets older to … determine exactly what they feel she might struggle with as she gets older.”—C2F2

Theme 2. Parents overall reported a lack of access to developmental and disability expertise, most often directing questions to therapists, other parents, or online resources	
2a. Parents primarily rely on therapists as sources of developmental information, but many also described other sources.	“I still talk to all of the therapists that she had when she was at [Children's Hospital] when I have questions about things, I'll email them, and they will help me, still… They let me email them.”—C2F2
	“I call here [Children's Hospital]. Most of the time, I speak with [nurse practitioner]. With any of my concerns, I just call up here, and they're willing to help at any time.”—C2F3
	“I think I'm going to ask her primary care doctor about her development.”—C2F9
2b. Parents often direct questions to parents of children with similar conditions.	“I feel like I keep asking. I would ask her nurse,—she has a trach baby too—…My question is, will she—I think it's every parent, right?—will she live a normal life?”—C2F8
	“There's a Facebook group for parents with this disease…So we get most of our information from other parents who have gone through it before.”—C1F2
	I: Is there someone now you would direct the questions that you have about him to? Not nitty‐gritty medical things, but bigger picture about his disability, who would you direct those questions to?
	D: I'd say Grandma.
	I: Mmhmm. She has been through this.
	M: And Grandfather, too, because they both went through it. My mother, because she was also a caregiver. Just those three.”—C2F9
2c. Parents are unsure of where to direct questions about delay and disability.	“I don't really have anyone to go to, when it comes to his disease specifically…So I would like to speak with someone—I know they mentioned someone at another hospital has kids with this case—I would like to speak with someone that has that knowledge about it.”—C2F10
	I: Do you have any expert or someone that you talk to about his overall development and disability?
	M: No, not at all.
	I: Would that be helpful, do you think?
	M: I think that might be.—C1F13
2d. Parents take initiative to find information and resources to support their children's development	“I always do my own research and stuff, and I know kids with Down syndrome take a little longer to do things, but they will get there. … I was on those Facebook groups.”—C1F22
	“But I just started looking it up after, so I can get my own knowledge about it.”—C2F10
	“I just want to make sure that she's getting everything that she needs, as she can be possibly getting it.”—C1F16
	“There's nothing much that we can do—if his lungs progress slowly, we can't do anything about it. But developmentally or strength‐wise, that's kind of our focus, is to try to help him to move along…And then you think as a parent, okay, maybe we're not doing enough. Maybe we need to do more.”—C1F11

*Note:* **Codes**: C, Cohort number; D, Father; F, Family number; I, Interviewer; M, Mother.

**Major Theme 1: Despite wanting the information and valuing development outcomes, parents report unclear expectations for delay and disability based on limited conversations with providers.**

*1A. Parents report limited prior discussions about their child's long‐term development and disability risks, often because inpatient conversations were focused on acute medical conditions*.

Many of the parents interviewed were unable to recall conversations with medical providers regarding their child's future development or disability risk. Parents noted that conversations in the hospital with the medical team focused more on acute medical issues and physical development which varied based on their child's medical conditions. “They haven't talked about anything concerning about her development … the only concern was her airway, and her gut.”

*1B. Most parents describe wanting to know about their child's risks of long‐term disabilities*.

In order to prepare and support their child's future, parents wanted to have more conversations about long‐term disability risks. One parent described, “it's better to know what's going on than to not to know anything. If we know everything… then we know how to prepare ourselves for any challenges.” Though parents acknowledge there can be a lot of uncertainty in prognostication, they desired more open communication and information about potential outcomes.

*1C. Parents do not have clear expectations for their child's developmental trajectory*

Parents often felt uncertain about their child's risk for long‐term disability. “I didn't know what to expect, I mean—you got a kid that's going through something, you don't know—I was thinking like he was going to have to be stuck in this bed, on the vent, all day, couldn't really play with his brothers and sisters?” Parents often anchored these expectations in terms of specific milestones or comparison to other siblings or peers but broadly felt unclear what their child's future looked like.

*1D. Parents who recalled developmental conversations with providers described them as vague and/or suggesting catch‐up potential*.

Recalled conversations with providers about development and disability were described as vague and not prognostic, with sentiments suggestive of full catch‐up potential, “they talked about delays in development and things that then sort of by kindergarten, she should be caught up with her typical peers.” When providers acknowledged delay, it was often without specific associated diagnosis, “They prepared us and warned us that he might be two to 3 months behind developmentally,—every single doctor tells you, “He's going to be behind.””

**Major Theme 2: Parents overall reported a lack of access to developmental and disability expertise, most often directing questions to therapists, other parents, or online resources.**

Some parents reported reaching out to inpatient or outpatient therapists when they had developmental questions or relying on online communities of parents. Generally, however, parents were unsure of where to turn for developmental expertise, had many questions about their child's developmental trajectory, and desired more information. “All the doctors—he's got his pulmonary, he's got cardiology, he's got endocrinology, he's got [Ear Nose and Throat]—and nobody can really answer my questions about his mental… everybody is concerned about his inner problems. But I wouldn't know who to talk to, actually!” Parents often noted trying to look up information on their own and find developmental resources for therapies or medical equipment. “We found out that he was behind, and we're going to jump ahead and do whatever we can, and use the resources that we can, to get him up and running.”

*Main Topic II: Parent'* *s Perspectives Development and Delay* (Table 3 )

**Table 3:** Topic 2—Detailed quotes for themes on parents’ perspectives on their child requiring invasive mechanical ventilation (IMV)'s development and developmental delays (*n* = 35).

Theme 1. Parents can identify specific developmental milestones and developmental delays in their children requiring IMV.	
1a. Many parents can identify gross motor milestones and delays, and these skills are frequent targets of intervention	“I think in general, it's that we need to get her to learn to sit on her own. I think that will be a big milestone for her and a big change for her.”—C1P15
	“Within the last week, she has started rolling almost onto her stomach, which is really pretty big. But that's pretty late, developmentally.”—C1F23
	“I: What do you think her greatest areas of delay are?
	M: Probably physical. Her physical movement. She's almost a year old, but she can't sit up.”—C1F16
	“My greatest concern is about her extremities, like how she can sit up, how she can crawl.”—C2F9
1b. Parents are concerned about their child's ability to eat by mouth and value acquisition of this skill.	“I think the only thing that is concerning to me is her trying to eat…I just feel like it's going take way longer to get her to eat now…she doesn't really like food. She doesn't really like too many things in her mouth besides toys… she's not used to anything soft in her mouth!”—C2F13
	“I'm hoping one day he's going to feed by mouth and the trach is off. He's going to eat independently on his own. That's what I want for him.”—C1F18
	“I would say my first goal is that she tolerates foods in general, because it's not always consistent. Sometimes she likes it. Sometimes she doesn't. I want to make sure that she gets to the point that I give her a spoon, and she's always responding favorably.”—C1F8
1c. Some parents identify deficits in communication and/or social skills	“A big worry of mine is he's not going to be able to communicate. I know it's going to be a while before he's going to be able to, you know, say sentences, or “mama,” “dada,” “I love you.” That's the stuff as a parent that you can't wait to hear—those first words.”—C2F7
	“I know for me, part of my concern is like, you know, peer interaction. He doesn't have access to peers or learning how to do social things really yet. Everybody is an adult around him.”—C1F12
	“The only thing I'm concerned with is that he doesn't respond to his name.”—C2F14
1d. When asked about development, many families share simple observations about improvements in their child's ability to engage with his/her environment and other people.	“She's just curious, the surroundings. Every day she's like finding new—something new she does…you can just tell she's more aware some days. Like she'll wake up more aware than she was the last day. So she's just more aware of different things.”—C2F13
	“She engages. She wants to learn about the environment and she's active, versus just laying in bed and not communicating with us.”—C1F5
	“She's also just becoming way more engaged in what's around her. She'll sit there and stare at herself in the mirror and smile at herself and kick all around and play, on her own, just happy as a clam. She'll interact—like she has a little stuffed animal bunny that sings songs and flips its ears around when it's singing songs. And she'll sit there and stare and giggle at that as it's playing.”—C1F15

**Theme 2. Parents have ideas about their child's overall picture of disability, sometimes attributing to specific diagnoses or components of medical complexity**	
2a. When asked to estimate the child's overall developmental age, parents often describe substantial gaps from biological age demonstrating their understanding of substantial delay.	“I: I know he's 33 months right now. How old do you think he acts?
	M: Like a newborn, I'll say.”—C2F4
	“I: He's 18 months if you correct. How old do you think he acts?
	A: I think he acts maybe six to eight months. Because I know he's starting to grab things…I think he's about six months old, or six to eight months, yeah.”—C2F7
	“I: Biologically, she's 16 months. How old do you think she acts?
	M: Maybe like—like a one‐year‐old.”—C2F13
2b. Parents often attribute delays in development to the presence of medical equipment (e.g. a tracheostomy).	“Well, for me personally— I'm worried about her eating and talking. Because the trach just more or less prohibits it, so that's a problem.”—C1F10
	“Since her trach is cuffed, and [child's name] has never been able to use her vocal cords since she has been born, my concerns is just her being able to, you know, speak. Even though I have hope that—I think that she will.”—C2F2
	“That's [ventilator] really the only thing that's holding him back. Because we haven't trialed him off, yet. I think once we do get the approval to start trialing him off, I don't think anything will hold him back!”—C2F5
	“I: What do you feel could help her eat better?
	D: Not having the tube. Not having the [tracheostomy].”—C2F6
2c. Parents acknowledge developmental delays, but attribute them to critical illness and long hospitalizations, often expecting them to resolve as critical medical illness improves.	“I also think she's very sleepy and fatigued because of all the medication she is on. Every day, we give her a lot of medicine.”—C1F21
	“She's obviously developmentally delayed. There's no way she couldn't be; she was in the hospital for almost eight months.”—C1F23
	“Since she has been in the hospital for a long time, and she was like sick, sick, sick… going for surgery… sleeping a lot, so her interaction is not that great.”—C2F7
	“She's behind, of course, because she had more or less a—she was basically confined to a bed for ten months. Even after she got the trach, last October—so she had more mobility, because she didn't have the breathing tube, of course. But she's still in the bed.”—C1F10
2d. Some parents describe specific diagnoses, most commonly describing risk or diagnosis of cerebral palsy and rarely describing risk of autism.	“Since [child's name] has shown early signs of cerebral palsy, that was one of my biggest concerns when she was preemie…As she got older, that's when they—I kind of saw it for myself, but I didn't want to claim it.”—C2F2
	“They told me like he might not walk, he might not talk, he might have cerebral palsy…He might be blind. He might not be able to hear. Just things like that.”—C2F14
	“I worry about autism. Because, you know, she was born at 27 weeks, and that puts them at higher risk…I have a lot of support groups, and a lot of the babies who were born super early, really early, a lot of parents are struggling with that. I just think—I was told that they are at higher risk.”—C2F8
2e. Some parents express no concerns about current developmental delay	“I: Do you ever worry about how he engages socially?
	M: No, not really.”—C2F7
	“I: Do you have developmental concerns about him?
	M: No, not at all.”—C1FI1

**Theme 3. Parents are hopeful about the future developmental trajectory of their child.**	
3a. Parents are encouraged by incremental developmental progress, especially in the context of medical fragility.	“Everything has been going well. She's progressed pretty much in every category like PT, OT, speech…it's all encouraging. None of the hardships matter when you see how well she's doing.”—C1F1
	“I think it's encouraging that we're seeing progress from him. It feels like we're kind of on the right track with what we're doing, and we see payoff with what we're doing …So yeah, we're doing OK.”—C1F4
	“Every single thing that he does is something they said he would never do. So, we sat around many tables where they said he wouldn't make it to the next day, and he did, every time… We're pretty amazed with how he's doing. I know he's delayed, of course, with everything he has gone through, but every day is just—he's learning. He's a little sponge, still. With such a tough beginning, it's pretty amazing.”—C2F1
	“Me as a parent, I know it's a little delayed, but I think he is learning every day, and I think that's really all that matters. He's doing new things every day, saying new things too. So, I think it's a slow process.”—C2F5
3b. Parents remain hopeful for ongoing developmental gains	“We're just hoping that everything will be fine, like we say. We don't mind how long it's going to take, but we just know that everything will be fine one day.”—C2F9
	“If, by a miracle, she could get off the ventilator and start talking, … I feel like she's going to be fine. Because like I said, her brain is fine. She's fine. She has everything available to her, to progress. It's not like her brain—I feel like when it's your brain, it's harder … I think she's going to be fine. I hope she's going to be fine.”—C2F8
	“I: a year from now, what do you think he's going to be like then?
	M: I think he's going to be caught up by then….I mean close to normal… I wouldn't say 100 percent because you can't expect that, but I would say close. Very close. Because he's on the right track.”—C2F14
3c. Some parents expect full developmental catch‐up	“I almost take offense when sometimes they try to teach her sign. I'm like, “Why? You don't need to teach her sign. She's going to be talking in a little bit. It'll be fine. We don't need to do sign and stuff.”—C1F10
	“M: She's already shown so much progress, so it's—I know she'll catch up….
	D: she has left us feeling very optimistic about her being able to catch up.”—C1F1
	“M: I don't know what the future holds, but I know she's going to do well. I know it's going to take time. She's going to catch up. I know she's delayed. But I know with time, with time, with—time will tell. But I know she's going to.”—C2F9
	“When she goes to school, she's going to be walking. She's going to be talking. She's going to be doing all these things. These other kids aren't going to know that she might not have walked until she was two or three. It's not going to make any difference. She's going to be just like any other kid out on the playground. It's just going to take her a different way to learn things. But she's just going to be able to do everything just like everybody else.”—C1F22

*Note:* Codes: C, Cohort number; D, Father; F, Family number; I, Interviewer; M, Mother.

**Major Theme 1. Parents can identify specific developmental milestones and developmental delays in their children requiring IMV.**

*1A. Many parents can identify gross motor milestones and delays, and these skills are frequent targets of intervention.*

Many parents noted delays in gross motor skills and identified these as the highest priority for habilitative focus. “His core and his strength physically are probably the areas for me that I'm most concerned about and really wanting to see more progress.” Gross motor skills were the most frequently discussed developmental domain; parents often focused on milestones such as head support, sitting, and walking.

*1B. Parents are concerned about their child's ability to eat by mouth and value acquisition of this skill*.

Parents valued eating by mouth and noticed this as an area needing habilitative focus. “The only area I'm really concerned about is eating. Because he is 2 years old, and he's not eating a thing.” Parents often described oral and/or feeding aversion as a barrier to eating by mouth even if they did not use that diagnostic term. “She doesn't really like too many things in her mouth besides toys.”

*1C. Some parents identify deficits in communication and/or social skills*.

Fewer parents noted concerns about delays in social skills and expressive communication. “He doesn't really communicate with us sign‐wise… so that's probably something I really want to work on.” Parents infrequently described communication and social skill concerns, and the majority did not link this observation to a unifying diagnosis.

*1D. When asked about development, many families share simple observations about improvements in their child*'*s ability to engage with his/her environment and other people*.

When asked open‐endedly about their child's development, some parents would share observations about their child's ability to engage with their environment and were reassured by their perception of progress as compared to how they interacted when in the hospital environment. “He laughs. He smiles. He claps his hands. He makes you feel like, yeah, we're getting somewhere.”

**Major Theme 2: Parents have ideas about their child's overall picture of disability, sometimes attributing to specific diagnoses or components of medical complexity.**

*2A. When asked to estimate the child's overall developmental age, parents often describe substantial gaps from biological age demonstrating their understanding of substantial delay*.

The interviewer frequently asked parents to estimate how old their child acts as compared to his/her biological age. Parents often gave a much younger developmental age, showing that they understood their child had substantial delays. “I: I know he's two and a half. How old do you think he acts? M: About one.” This demonstrates a general understanding and acknowledgement that substantial delays exist for their child even for families who did not discuss specific diagnoses of global developmental delay, intellectual disability, or other neurodevelopmental disability.

*2B. Parents often attribute delays in development to the presence of medical equipment (e.g. a tracheostomy)*.

While parents were often able to identify delays, many parents attributed these to the presence of medical equipment. One parent noted, “If he didn't have these tubes on him holding him back, he'd have taken off like a rocket.” The presence of the tracheostomy specifically was often a reason parents gave for delays in speech and feeding. Some parents articulated an expectation that the delay would resolve when the medical equipment was removed.

*2C. Parents acknowledge developmental delays, but attribute them to critical illness and long hospitalizations, often expecting them to resolve as critical medical illness improves*.

Other parents noted the long hospitalization or presence of medical illness as factors contributing to delays, however, expected these delays to resolve as their child became more medically stable. “Because it wasn't his heart condition stopping him from breathing. It was more of his undeveloped lungs. All of this stuff can go away.” Families often described improvements in development and engagement after hospital discharge. This progress reinforced their expectations for developmental catch‐up as medical fragility improved.

*2D. Some parents describe specific diagnoses, most commonly describing risk or diagnosis of cerebral palsy and rarely describing risk of autism*.

Most parents discussed developmental concerns and potential generally, however a few parents specifically articulated an increased risk of cerebral palsy, “Because they mentioned to us at the hospital that she may have cerebral palsy, but that they didn't know if it would affect her because she was too young. That they didn't know whether she could see, hear, speak or eat on her own or walk.” Only a few parents directly mentioned risk of autism spectrum disorder.” I've been looking into autism … I'm not a doctor, but I know my baby, and I see certain things that she does.”

*2E. Some parents express no concerns about current developmental delay*

Finally, some parents expressed no concerns about their child's development at all. “Even though he's not walking yet, I actually have no concerns.” The interviewer often probed in specific areas of development after an initial open ended question; a subset of parents denied both global and specific developmental domain concerns.

**Major Theme 3: Parents are hopeful about the future developmental trajectory of their child**

*3A. Parents are encouraged by incremental developmental progress, especially in the context of medical fragility*.

Parents were encouraged by their child's progress since discharge and many expected it to continue, especially in the context of medical fragility and delays during hospitalization. “But I guess overall she's doing well for what I expected her to be, considering everything she's been through.”

*3B. Parents remain hopeful for ongoing developmental gains*

Looking forward, parents are hopeful for ongoing developmental gains and often describe this in the context of putting in the work and effort in order to achieve continued progress. “[She's] not where [she] needs to be, but just sort of behind, and hopefully they'll be able to help us help her get there”. This “work” was sometimes meaning parental effort, support from therapists, or the resilience and character of their child themselves.

*3C. Some parents expect full developmental catch‐up*

Many parents who did acknowledge their child exhibited current developmental delays discussed their expectation for developmental catch‐up to “normal,” without future delays. “And no doubt, he'll catch up. I have no doubt in my mind that this kid is so smart, and he will figure it out.”

## 4 Discussion

Children requiring IMV have a high likelihood of manifesting developmental delays and disabilities, and therefore, parents of children requiring IMV ought to be poised with the tools to support their habilitative needs, including the knowledge of their neurodevelopmental risks. Through in‐depth interviewing, we explored parents’ recall of prior conversations with healthcare providers and their own perceptions about their children's development. We discovered that while parents are having many conversations and interfacing with a variety of providers during the care of their hospitalized child, they recalled conversations as rarely focusing on delay or disability risks. Despite this, most parents have a general sense of their child's overall delay. Many parents describe an expectation for full developmental catch‐up, and rarely did parents mention specific diagnoses known to impact premature survivors and children requiring IMV at high rates. To our knowledge, this study is the first to investigate the perspectives of parents of children requiring IMV on development and developmental counselling.

Parents recall having had few conversations directly addressing disability risks, despite frequent and in‐depth experiences with healthcare systems and providers. They described that conversations while inpatient were focused on acute medical conditions and training in anticipation of discharge. Given the long hospitalizations of children requiring IMV, there may be missed opportunities for counseling on disability risk which incorporates prognostication based on the child's specific complexity and developmental assessments. Parents described not knowing who to direct questions to and wanting more information about their child's risks and trajectories. Ultimately, this study revealed that despite long hospitalizations and many interactions with healthcare professionals, conversations about disability risk are scant and do not match parental desire for more information.

Even though parents describe not having direct conversations, they are often able to identify their child's developmental delays and often had a sense of substantially delayed development. Many parents focused on gross motor skills, and some parents also identified differences in social communication or feeding milestones. Despite most parents observing delays, parents were generally encouraged by their children's incremental progress and were hopeful for catch‐up. Parents acknowledged that developmental progress would require a lot of effort and engagement, and they were eager to support this, often seeking more resources and information. Many families described that they expected their child to fully catch‐up to same age peers.

Future work to shape anticipatory guidance for parents may consider existing literature and practices around pre‐delivery counseling, when in many settings, parents anticipating the delivery of a premature baby receive focused counseling on survival and disability risk based on the child's anticipated gestational age. Studies of these discussions have been found to be most accurate when gestational age corresponds with clear guidelines for counseling, but overall conversations have been found to focus disproportionately on short‐term risks, overlooking long‐term problems [28]. The effectiveness of this pre‐delivery counseling is mitigated by high levels of anxiety in birthing parents, variability in physician knowledge, and communication gaps between providers and parents [29, 30, 31, 32]. Similarly, guidance for parents of children requiring IMV should consider timing of conversations which may differ from family to family.

Overall, our study suggests that parents desire more knowledge, conversations, and access to expertise to understand developmental prognosis of their children. Parents in our study were consistently hopeful and motivated to intervene to best support their child's development. Specific comments from parents in this study suggest that counseling from providers ought to be direct, yet hopeful, and reference specific disabilities.

These results should be interpreted in light of several important limitations. First, the inherent nature of retrospective interviewing is that it is subject to recall bias. Parents themselves acknowledge how overwhelming and foggy early conversations during their child's hospitalizations were, consistent with prior literature on NICU counseling [33]. Timing conversations about disability risk ought to be considered, as well as how information is delivered (written in addition to verbal communications, e.g.) and needs differ for each family. Additionally, our sample was limited to children within a single region; our conclusions may not be generalizable to other locations which have different discharge or counseling processes. Despite this limitation, these in‐depth individual interviews shed light on a previously understudied topic.

Children requiring IMV are a broadly understudied population and greater knowledge about long‐term developmental risks are needed. Future investigations comprised of larger cohorts followed with neurodevelopmental assessment would guide this risk and prognosis counseling. Other studies ought to investigate provider opinions on developmental prognostication and counseling to determine potential drivers of the limited conversations parents described. Ideally, parent and providers ought to work together to develop best approaches to these sensitive conversations. Additionally, this study revealed that therapists are critical players in supporting and providing developmental guidance, and a trusted resource to families, yet not poised to disclose diagnoses. The role of these critical partners for supporting neurodevelopment ought to be explored in concert with other members of complex care teams.

## 5 Conclusions

Parents of children requiring IMV desire honest disclosures about disability risk but infrequently recall having had such conversations. Without using specific diagnoses, families often identify their children as having substantial developmental delays. Future studies ought to explore provider perspectives about developmental trajectories, disability risks, and counseling conversations. Ultimately, improving communication between providers and parents will best support children requiring IMV and their families in preparation for potential long‐term disabilities.

## Author Contributions

Amanda Calipo participated in data coding, data analysis and interpretation, drafting and revising the manuscript. Emma L. Green participated in subject recruitment and study coordination, data analysis and interpretation, drafting and revising the manuscript. Iris Huang participated in data coding, data analysis, and revision of the manuscript. Sarah A. Sobotka participated in study concept and design, participant interviews, data coding, data analysis and interpretation, writing the initial paper draft and critical revision of the manuscript. All authors approved the final manuscript as submitted and agree to be accountable for all aspects of the work. An abstract of this work was presented at the 2024 Pediatric Academic Societies Meeting and an oral free paper presentation was given at the 2024 American Academy for Cerebral Palsy and Developmental Medicine Conference.

## Ethics Statement

This study was approved by the University of Chicago Institutional Review Board (IRB 17‐0908 and IRB 21‐1075) and informed consent was obtained from all participants.

## Conflicts of Interest

The authors declare no conflicts of interest.

## Data Availability

Data that support the findings of this study such as interview transcripts cannot be made publicly available in order to preserve anonymity of families interviewed. De‐identified quote tables from the analytic process may be made available upon reasonable request to the authorship team.
